# Characterization of signal and transit peptides based on motif composition and taxon-specific patterns

**DOI:** 10.1038/s41598-023-42987-1

**Published:** 2023-09-21

**Authors:** Katarzyna Sidorczuk, Paweł Mackiewicz, Filip Pietluch, Przemysław Gagat

**Affiliations:** Department of Bioinformatics and Genomics, Faculty of Biotechnology, University of Wrocław, Wrocław, Poland

**Keywords:** Protein sequence analyses, Chloroplasts, Endoplasmic reticulum, Mitochondria, Protein translocation

## Abstract

Targeting peptides or presequences are N-terminal extensions of proteins that encode information about their cellular localization. They include signal peptides (SP), which target proteins to the endoplasmic reticulum, and transit peptides (TP) directing proteins to the organelles of endosymbiotic origin: chloroplasts and mitochondria. TPs were hypothesized to have evolved from antimicrobial peptides (AMPs), which are responsible for the host defence against microorganisms, including bacteria, fungi and viruses. In this study, we performed comprehensive bioinformatic analyses of amino acid motifs of targeting peptides and AMPs using a curated set of experimentally verified proteins. We identified motifs frequently occurring in each type of presequence showing specific patterns associated with their amino acid composition, and investigated their position within the presequence. We also compared motif patterns among different taxonomic groups and identified taxon-specific features, providing some evolutionary insights. Considering the functional relevance and many practical applications of targeting peptides and AMPs, we believe that our analyses will prove useful for their design, and better understanding of protein import mechanism and presequence evolution.

## Introduction

The localization of a protein within a cell is of crucial importance for the correct functioning of both the protein and the cell. The information about protein destination is hidden in its amino acid sequence as a targeting peptide or in its structure^1^. The most well-known targeting peptides are N-terminal extensions of proteins. They address proteins to their proper subcellular compartments and are usually cleaved off during the transport, resulting in the release of mature protein^2^. The most common targeting peptides are signal peptides (SPs) and transit peptides (TPs), hereafter also collectively referred to as presequences.

SPs allow protein translocation through the endoplasmic reticulum membrane, which is a prerequisite for further transport, including secretion outside the cell^3^. They occur universally in all taxonomic lineages. Generally, three structural components of SPs are distinguished in the following order: (i) the n-region characterized by the presence of positively charged residues, (ii) the h-region rich in hydrophobic amino acids, and (iii) the c-region, slightly polar, containing a cleavage site^2^. The net positive charge of the n-region has been shown to differ between groups of organisms, e.g. the Gram-positive bacteria have more charged residues than the Gram-negative species^4^. Additionally, the positive charge in the n-region has been demonstrated to be especially important for the translocation of small eukaryotic secretory proteins^5^. The hydrophobicity of the h-region is crucial for entering the lipid bilayer, and its motifs are also species-specific, with leucine being the most frequent amino acid and the LLL sequence often found in eukaryotes^6^. The c-region is short and composed of neutral or polar amino acids, with alanine residues found most frequently at the first and third position proceeding the cleavage site; however, other neutral amino acids are also tolerated here, especially in eukaryotes^2,6^.

TPs enable the translocation of nuclear-encoded proteins into the organelles of endosymbiotic origin, i.e. mitochondria and chloroplasts, through their envelopes. Chloroplast transit peptides (cTPs) are considered the most diverse group of organelle targeting peptides in terms of primary sequence and lengths, but they share a few general characteristics, such as an increased number of serines, decreased number of acidic residues and moderate hydrophobicity in their N-terminal part^7,8^. They also contain numerous proline residues, which are suggested to keep cTPs in an unstructured form and facilitate subsequent steps of protein import^9^. Mitochondrial transit peptides (mTPs) are evolutionarily older than cTPs, preceding them by hundreds of millions of years^10^. They tend to be shorter than cTPs and possess an increased number of arginine, especially within the 12 first residues, as well as leucine^11,12^. Noteworthy, the leucine-arginine dipeptide has been shown to be important for the mTPs prediction^13^. The cationic charge generated by arginine is specifically used for the import of mitochondrial matrix proteins across the electrochemical gradient of the inner mitochondrial membrane^14^. Mitochondrial TPs are also characterized by the presence of φ_ _φφ motif, where φ corresponds to a hydrophobic or aromatic amino acid. This particular motif was suggested to function as a Tom20 recognition site^15^.

Being connected to one of the most important evolutionary processes, i.e. endosymbiosis, the origin of TPs has resulted in the formulation of many hypotheses^16–18^. One assumes that TPs evolved from antimicrobial peptides (AMPs), short molecules providing organisms with the defence against microorganisms^19^. AMPs are mostly cationic, hydrophobic as well as amphipathic, and consequently possess a natural ability to be attracted to negatively-charged bacterial cell membranes. This hypothesis assumes that AMP genes merged with genes encoding organelle-targeted proteins and thereby facilitated their import into bacteria-derived organelles^19,20^.

Until now, presequences have been studied in terms of their amino acid composition, physicochemical properties or specific motifs. Additional studies involved prediction of certain presequences or analyses of cleavage sites^13,21^. Experimental studies focused on TPs resulted in the identification of several amino acid motifs crucial for protein import, e.g. SSXXSS motif in cTPs or VVRNR motif in mTPs^22^. However, the majority of experiments were based on individual proteins from the thale cress (*Arabidopsis thaliana*) or pea (*Pisum sativum*)^23,24^; therefore, the presence or importance of such motifs in other proteins or organisms remains questionable. Some of the identified motifs are semi-conserved, e.g. FGLK motif of cTPs has many variants: FNGLK, FLRKQP and FPVKK^25^. Other motifs are not very specific, e.g. MHSM, which stands for moderately hydrophobic sequence motif, and MAR, which translates into multiple arginine residues, both found in mTPs^26^. Studies in silico allow us to gain more general insights into presequences, e.g. their amino acid composition and amino acid patterns visualized in the form of sequence logos^7,27,28^. Moreover, it has been shown that cTPs and mTPs can be distinguished based on certain physicochemical properties^29^ and some functional motifs have been identified in the h-regions of SPs across different species^30^. However, none of the previous studies has provided a complete overview of amino acid motifs in these targeting peptides.

Here, we present a global analysis of the motif composition of presequences using a curated set of experimentally verified proteins. In contrast to previous studies, we started by identifying all motifs occurring in each type of presequence using a memory-efficient method of k-mer (i.e. short sequences) counting. It allowed us for detailed analyses of a whole landscape of motifs of different sizes. We performed a comprehensive research of amino acid motifs in targeting peptides as well as identified common motifs in each presequence type and their positioning. We also explored motif patterns among different taxonomic groups and identified taxon-specific motifs, thereby contributing important evolutionary information. Overall, this work is the first, to our knowledge, approach to the global analysis of motif content within targeting peptides, allowing to define characteristics of each presequence considering taxonomic diversity and specificity. It is worth mentioning that we investigated AMPs in this context as well. Furthermore, our results provide insights useful for the design of targeting peptides and AMPs, which show many promising applications, e.g. in biotechnology and medicine^6^.

## Results

### Most frequent motifs in targeting peptides

We considered motifs composed of two to five specified amino acids, including discontinuous (also called gapped) motifs, i.e. containing an unspecified amino acid in some positions and, for clarity, represented in the motif pattern by the lower dash symbol ‘_’. For bigrams and trigrams (motifs of two and three specified amino acids, respectively), we considered one to three unspecified amino acids; for tetragrams (motifs of four specified amino acids) one to two unspecified amino acids; for pentagrams (motifs of five specified amino acids) only one unspecified amino acid between two specified residues.

**Figure 1:**
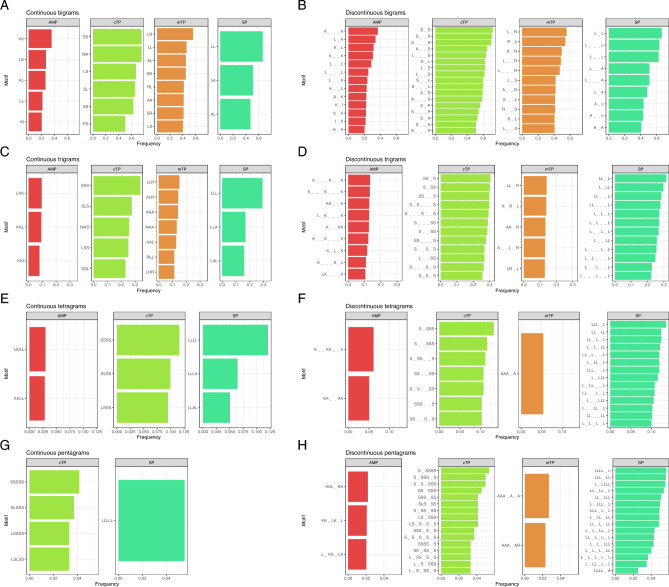
The most frequent motifs in targeting peptides and AMPs. Frequency cutoffs have been chosen for each sequence type separately due to the large differences. The lower dash symbol ‘_’ indicates an unspecified amino acid in the motif. Data used for generation of plot is available as Supplementary Data.

Chloroplast TPs are characterized by the occurrence of motifs with a very high frequency compared to other presequences, with motifs SS, MA, S_S and S_·_S found in over 70% of cTPs (Fig. 1). Bigram frequencies indicate the presence of serine, alanine, leucine and proline in over 50% of cTPs. With the increasing length of the considered motif, the general prevalence of serine-rich motifs, sometimes with the addition of leucine, becomes apparent. Noteworthy, over 10% of cTPs possess a stretch of four consecutive serines or motifs composed of four serines in combination with other amino acids. Some of these motifs may occur multiple times in the same sequence, especially SS and A_·_S, which are present twice in over 20% of cTPs. The highest number of repeats in a single cTP was observed for motifs: A_·_A (up to 11 repeats), SS and S_S (up to 10 repeats), AA and A_A (up to 8 repeats).

In the case of mTPs the most frequent motifs are present in over 40% of sequences and are composed of leucine, arginine, alanine and serine, with LR, L_R and R_L motifs occurring in over 50% of mTPs (Fig. 1). Longer motifs occur with a lower frequency and are characterized by the higher content of alanine residues. For example, trigrams: ALR, AAA, MAA, AAL, A_R_L, AA_R and A_·_L_R are found in 10–15% of mTPs. Interestingly, mTPs do not possess longer motifs with high frequency, as only a single tetragram AAA_A is present in more than 5% of analysed sequences (Fig. 1). L_R, L_·_R, R_L, L_·_S, A_R, LR and A_A motifs occur twice in more than 10% of mTPs. Motifs with the highest number of multiple occurrences include: AA (up to 10 repeats), SS and A_A (up to 9 repeats), NN and AAA (up to 8 repeats).

SPs show a strong preference for leucine-containing motifs, with LL and LLL present in over 65% and almost 30% of SPs, respectively (Fig. 1). Even longer stretches of leucine are relatively common, as four and five consecutive leucine residues are found in over 12% and 5% of them, respectively. Noteworthy, most frequent motifs: LL, LA, L_L, L_·_·_L, L_·_L, L_·_A and L_·_·_A are composed exclusively of leucine or leucine and alanine as specified amino acids, and they were found in over half of the analysed SPs. Leucine motifs also occur multiple times in the same presequence, especially LL, L_L and L_·_L, which are found twice in more than 20% of SPs; three repeats of LL are present in 10% of SPs. Interestingly, the motif with the highest number of repeats is A_·_A (up to 13 repeats), followed by AA and A_A (up to 11 repeats), LL (up to 10 repeats), as well as P_P, L_L, S_S and LLL (up to 9 repeats).

AMPs are characterized by motifs composed of lysine and hydrophobic amino acids such as leucine, isoleucine and alanine. The most frequent motifs are: KK and K_·_·_K found in over 35% of sequences, followed by L_K, K_L, K_·_K, L_·_L, LK and KL occurring in more than 25% of AMPs (Fig. 1). We identified only two tetragrams, i.e. K_·_KK_·_K and KK_·_KK, present in more than 5% of sequences. However, the AMP set was the most numerous with almost 10,000 sequences, which could influence the frequency values. AMPs are also characterized by a higher number of repeats of individual motifs within the same sequence, e.g. K_·_K (up to 40 repeats), K_·_K_·_K (up to 38 repeats), K_·_·_K_·_·_K and K_·_K_·_K_·_K (up to 36 repeats), P_P (up to 25 repeats), and LK (up to 24 repeats).

### Positional specificity of common motifs

**Figure 2:**
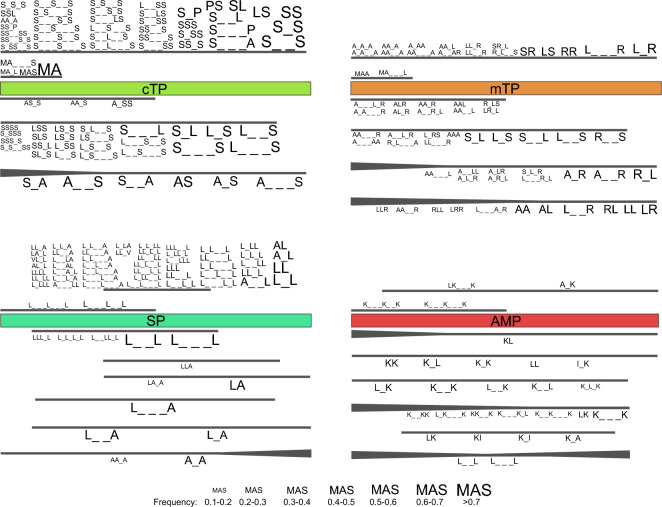
Positional specificity of motifs frequently occurring in targeting peptides and AMPs. Coloured rectangles represent the whole length of a given type of presequence or AMP. Black lines above or below the rectangles mark specific parts of the sequence in which most frequent motifs were found. The widening of the black lines means that the motifs are more likely to occur in this region. The size of the motifs corresponds to their frequency, as indicated in the legend. The lower dash symbol ‘_’ indicates an unspecified amino acid in the motif.

We noticed a non-uniform distribution of the most frequent motifs along sequences of investigated targeting peptides and AMPs (Fig. 2). What stands out is the variety of motifs found along the whole length of both cTPs and mTPs, whereas the majority of motifs present in SPs occur in their middle part. AMPs generally possess fewer motifs with high frequency, and they are usually spread along the whole sequence; only two motifs were identified in the first part of AMPs.

TPs possess motifs specific to their N-termini, particularly conserved among cTPs, as over 75% of these presequences start with MA. Longer N-terminal motifs occurring in more than 10% of cTPs include: MAS, MA_L and MA_·_·_S. Similarly, mTPs often contain MAA or MA_·_·_L at their N-termini. Both types of TPs also possess some motifs that are present mainly in the first part of the presequence. These motifs are composed of serine and alanine in cTPs (A_SS, AS_S, AA_S) and alanine, leucine, arginine and serine in mTPs (e.g. LR_L, AL_R, AA_R, R_LS, etc.). Both types have a lot of motifs that are spread with relatively similar prevalence along the whole sequence, e.g. SS, LS, S_S, S_·_S in cTPs and LS, SR, RR, L_R in mTPs. Interestingly, TPs share some motifs that do not occur near their C-termini, e.g. L_S, S_L, L_·_S, and also some motifs that characterize the whole sequence but are most frequently placed near or in their N-terminal part, such as: AS, A_S, S_A, A_·_S, S_·_A, A_·_·_S in cTPs, and LL, LR, RL, R_L, L_·_R, AA, A_R, A_·_R, among others, in mTPs (Fig. 2).

The most frequent motifs of SPs show the highest specificity for the middle part of the presequence, where also the majority of motifs occur. These are mainly leucine-rich motifs, e.g. LL, L_L, L_LLL, LLL_·_L, or motifs comprising leucine with the addition of alanine and valine as defined amino acids, e.g. AL, A_·_L, LL_V and VL_L. Two motifs show selectivity towards the first part of SPs, i.e. L_·_·_L_·_ L and L_·_·_L_·_·_ L, and a few are spread along the whole presequence, e.g. L_A, L_·_A, L_·_·_A. In contrast to TPs, SPs possess some motifs found along the whole sequence but more frequent at the C-terminus, i.e. A_A and AA_A (Fig. 2).

According to our results, motifs KK, LL, K_L, K_K and I_K are distributed relatively uniformly in AMPs. Others, e.g. L_K, K_K, L_·_K and K_L_K, do not occur at the C-termini. A_K and LK_·_·_K may be present anywhere except the N-terminus, whereas LK, KI, K_I and K_A are not found at both N- and C-termini. KL is present along the whole AMP sequence but most frequently at their N-termini. Two motifs, K_·_·_K_·_K and K_·_·_K_·_·_K, occur in the first part of AMPs, whereas others, e.g. LK, K_·_·_K and KK_·_K, are most frequent in the first part but can be find anywhere in the sequence except for the C-terminus. An interesting localization pattern, not seen in other targeting peptides, is exhibited by L_·_L and L_·_·_L motifs, which are most prevalent at the N-terminus and in the second part of the AMP sequence but not C-terminus (Fig. 2).

### Application of reduced alphabets on targeting peptides

In order to make the motifs more general, we reduced the 20-letter amino acid alphabet to a smaller number of elements grouping amino acids with specific physicochemical properties. Next, we searched motifs in presequences recoded in this way.

Using the alphabet assuming seven amino acid groups (enc7), we identified as many as three continuous, i.e. [AVIL][STNQ][STNQ], [AVIL][STNQ][AVIL] and [STNQ][AVIL][STNQ], as well as 18 gapped trigrams in more than 75% of cTPs (Figs. S1 and S2). Noteworthy, all these motifs are combinations of two groups of amino acids, i.e. hydrophobic: A, V, I, L and polar uncharged: S, T, N, Q. The encoding in which serine and threonine represented a separate group (enc8) resulted in two discontinuous ([AVIL][ST][AVIL], [AVIL][ST][ST]) and one continuous ([AVIL]_[AVIL][ST]) trigram with the frequency over 70%, all comprising serine/threonine and small hydrophobic amino acids (Figs. S3–S4).

In the case of mTPs, the use of enc7 and enc8 alphabets produced six motifs found in more than 65% of sequences, i.e. one continuous ([AVIL][AVIL][RHK]) and five gapped trigrams, all consisting of only hydrophobic amino acids or with the addition of a positively charged amino acid (R/H/K) (Figs. S1–S4). The encoding enc10 revealed that arginine occurs most frequently in these motifs, with [VIL]_R_[VIL] and [VIL][VIL]R present in over 30% of mTPs (Figs. S5–S6).

The application of enc7 and enc8 alphabets on SPs resulted in motifs with very high frequencies due to their preference for hydrophobic residues. We obtained 11 trigrams (one continuous, ten discontinuous) composed exclusively of hydrophobic amino acids: alanine, valine, isoleucine and leucine, occurring in more than 90% of SPs (Figs. S1–S4). Even tetragrams show high frequencies, with seven gapped motifs present in more than 75% of SPs. Interestingly, these encodings revealed one novel pattern that did not appear on the whole alphabet, i.e. motif [AVIL][AVIL][CGP] present in over 55% of SPs. Applying encoding with more groups, i.e. enc10, indicated some novel patterns with large aromatic and hydrophobic amino acids, however with lower frequencies, e.g. [VIL][FYW][VIL] and [FYW][VIL][VIL] present in approximately 25% of SPs (Figs. S5–S6).

### Taxon-specific motifs in targeting peptides

**Figure 3:**
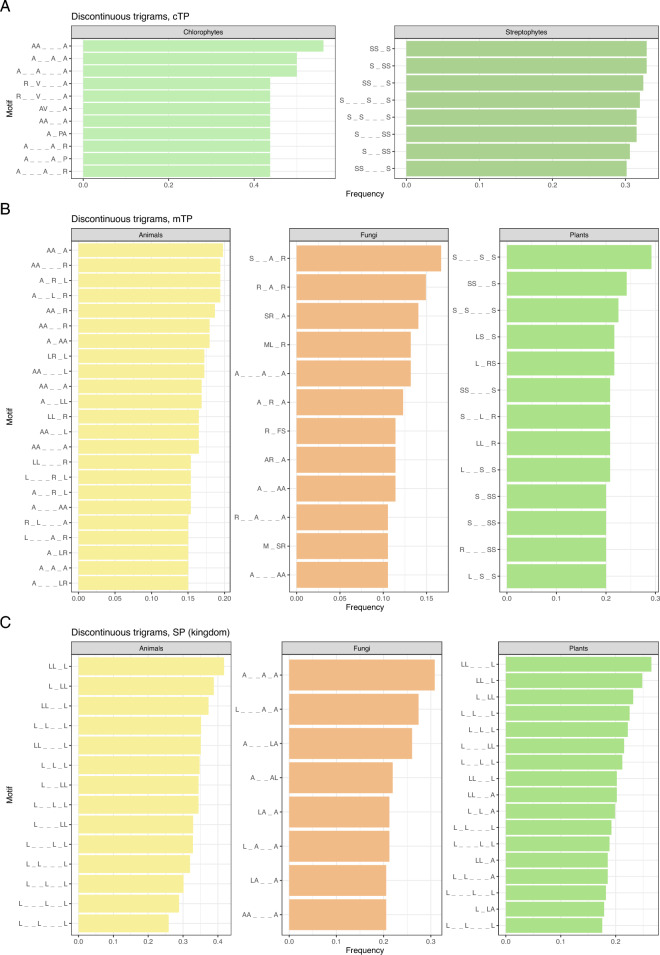
Taxon-specific trigrams for cTPs (**A**), mTPs (**B**), and SPs on kingdom level (**C**). Other types of motifs and comparisons of different taxonomic levels for all targeting signals are provided in Figs. S8–S13. The lower dash symbol ‘_’ indicates an unspecified amino acid in the motif.

**Figure 4:**
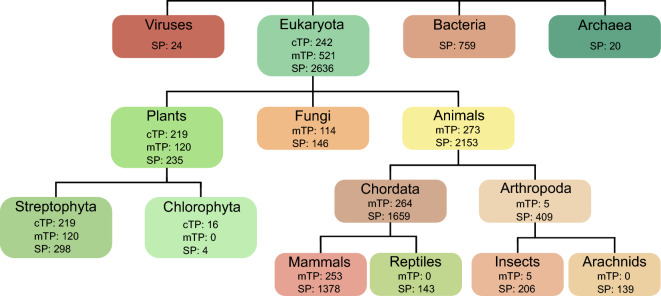
Number of presequences within each investigated taxonomic unit. Only the taxa used for visualization of results are shown; for the full list of taxa and their corresponding sequence counts see Table S3. The number of sequences in the higher taxonomic levels may not sum up to the sequences of lower levels as only the selected taxa are presented.

For cTPs, some motifs are frequently found in both Streptophyta and Chlorophyta, i.e. MA, AS, A_S, A_·_S, A_·_·_S, S_A, occurring in at least 50% of sequences with no statistical differences between them (Figs. S7–S8). Other motifs with no significant differences are e.g. AA, SR, R_S, S_·_A, S_·_·_A, MAS and ASSS. Motifs that are most frequently found in streptophytes and significantly less often in chlorophytes include e.g. SS, LS, ST, PS, L_S, L_·_S, S_P, S_S, SSS, SSL, SS_S, S_SS; and motifs: SP, T_·_S and SLS were not identified in chlorophyte cTP at all (Figs. 3, S8). In turn, motifs found significantly more often in Chlorophyta are e.g. A_R, V_A, A_·_A, A_·_·_A, A_·_·_R, PA, A_A, R_A, R_·_A, R_·_V, R_·_·_A with frequency over 70%. The differences are much more visible for longer motifs. Many of them occur exclusively in one taxonomic group, e.g. SLS and SSSS in streptophytes and PAVR, KASA, I_A_·_A_·_A and F_P_·_R_·_A in chlorophytes, all with frequencies higher than 15% (Fig. S8). However, it is important to note that chlorophytes represent a taxon in our dataset with the lowest number of available sequences (Fig. 4).

Analysis of mTP motif distribution between taxonomic groups covered comparisons among three traditional kingdoms, i.e. plants, animals and fungi. Motifs significantly more often found in plants included: SR, RS, RR, SS and S_S with a frequency of at least 60%, S_L, S_R, S_·_S, S_·_L with a frequency over 50%, as well as SSS, LRR, SS_·_S, S_·_·_S_S, LS_S, L_RS and L_·_S_S with frequency at least 20% (Figs. 3, S9). Animals differ from plants and fungi mainly in the frequency of AL, AA, R_L, A_A and A_·_R, which are present in over 50% of animal mTPs, as well as MAA, AAL, AAA, AA_R, AA_A, AA_·_ R, A_R_L, A_·_L_R, LLL_L and LL_LL occurring in more than 17.5%. Motifs found significantly more often in fungi are ML with frequency over 40%, along with S_·_A_R, SR_A, R_A_R, ML_R, MLS and MLR found in more than 12.5% of fungal mTPs (Fig. S9).

SPs show a few differences in motifs among the three domains of life (Archaea, Bacteria, and Eukarya) and also viruses. For example, GA, M_·_S, L_T and A_·_G occur significantly more often in archaeons with a frequency of at least 50% (Fig. S10). Other motifs specific to Archaea include: A_·_LA, GA_A and A_·_L_·_L, found in 40% of SPs, as well as other motifs rich in: leucine, methionine, valine, glycine and alanine. Viruses are characterized by VLS and L_·_VL, present in over 20% of sequences (there was no statistical significance between viruses and archaeons due to the low number of sequences), and the high frequency of LLLL motif, which also often occur in eukaryotes. Motifs most commonly present in bacteria are composed mainly of alanine and leucine, sometimes with the addition of serine and glycine. Eukaryotic SPs are characterized by motifs composed exclusively of leucine as a specified amino acid and less frequently leucine with alanine or glycine. Their strong preference for leucine-rich motifs is clearly visible for longer sequences, as all frequently occurring gapped trigrams and tetragrams consist of leucine. Moreover, LLL is present in 35% of eukaryotic SPs, whereas LLLL and LLLLL in over 15% and 7%, respectively (Fig. S10).

The division of eukaryotes into traditional kingdoms shows distinct differences between plant, animal and fungal SPs. Plants are characterized by the MA motif present in more than 50% of SPs. Other motifs significantly distinguishing plants include FLL and L_LFL, found in over 15% and 6%, respectively (Fig. S11). Animals are characterized by the presence of leucine-rich motifs with the highest frequency of LL, L_L, L_·_L and L_·_·_L, occurring in more than 70% of SPs (Fig. 3). All gapped trigrams and longer motifs with the highest frequency are composed exclusively of leucine as a specified amino acid (Fig. S11). Moreover, stretches of three, four and five consecutive leucines are present in over 25%, 17% and 8% of animal SPs, respectively. Motifs that appear significantly more often in fungi include e.g. LA, AL and AA, found in over 50% of SPs; as well as V_A, A_A and A_·_A, found in more than 40%; and ALA in 30%. Longer motifs often present in fungi usually comprise: leucine, alanine and valine.

Further differentiation of animal SPs into chordate and arthropod presequences revealed the most distinctive difference in frequency for motifs: LL (82% in Chordata vs. 52% in Arthropoda), LLL (45% in Chordata vs. 18% in Arthropoda) and LLLL (21% in Chordata vs. 4% in Arthropoda) (Figs. 4, S12). Generally, motifs composed of leucine are characterized by much higher frequency in chordates. Arthropods, besides leucine motifs, contain some with leucine and valine, but they also occur in chordates quite frequently. Chordate SPs seem to consist of mainly leucine-only motifs, i.e. leucine is the only specified amino acid, or those with leucine and alanine or to a lower extent valine. On the other hand, arthropods SPs do not possess these motifs occurring with that high frequencies but most frequent for this group are motifs of leucine and valine (Fig. S12).

We also investigated the distribution of motifs in SPs among: arachnids, insects, reptiles and mammals (Figs. 4, S13). Arachnids are characterized by isoleucine-containing motifs, e.g. LI, I_·_·_L, L_·_I, L_·_·_I and I_L, found in more than 40% of SPs. Insects possess motifs mainly composed of leucine, alanine and valine e.g. LV, VL, V_A, L_A, L_·_·_A, but they also occur in other groups. Reptiles have a higher frequency of motifs combining leucine with valine or glycine, e.g. V_L, V_·_·_L, L_V, L_·_V, L_G, present in over 50% of SPs. Much overrepresented motif in reptiles is TL, which is found more than twice as frequently in this group compared to the other analysed (almost 50%). Mammals have the highest frequency of motifs composed exclusively of leucine as a defined amino acid, with L_L, L_·_L and L_·_·_L occurring in more than 80% of SPs. Longer motifs emphasize the preferences of taxonomic groups towards specific amino acids besides leucine, i.e. isoleucine and valine in arachnids; alanine and valine in insects; glycine, valine, threonine and cysteine in reptiles; and only a slight addition of alanine in mammals. Interestingly, reptiles are characterized by the presence of many long and complex motifs occurring with high frequency, e.g. TLLLT, TIVCL, MKTLL, LSLSG, KTLLL, CLDLG, found in more than 15% of SPs, as well as V_·_SL_G, T_·_CLD, M_T_LL, LL_L_·_V, present in over 20%. Moreover, as many as 16 continuous tetragrams occur in at least 15% of reptilian SPs.

## Discussion

Presequences and AMPs represent groups of heavily studied peptides due to their functional properties and auspicious applications^6,31^. Nevertheless, current studies provide insufficient information about important sequence motifs as experimental approaches are expensive, time-consuming and focus on individual sequences, e.g. the small subunit of RuBisCO or chlorophyll a/b-binding protein from *Arabidopsis* in the case of the cTPs^23^. Moreover, they usually focus on one type of presequence, making comparisons between different targeting peptides difficult. Our in-depth analysis of motif composition of presequences and AMPs resulted in: (i) the identification of their most frequent motifs, (ii) indication of their positioning within the sequence and (iii) revealing taxon-specific patterns within each peptide group. The results allow us to revise the definition of each presequence and to verify if the motifs described in the literature for individual sequences transfer into a broader context covering multiple taxonomic lineages.

For a long time, cTPs were considered to be abundant in hydroxylated amino acids, until a more comprehensive study of *Arabidopsis* and rice proteins revealed that the former is enriched in serine, while the latter in alanine and none in fact in threonine^32,33^. Here, we take a step further and report that serine-rich motifs are the most distinctive feature of cTPs. They also often include leucine, alanine and less frequently proline. Moreover, serine-rich motifs seem to be especially characteristic of streptophytes, whereas chlorophytes possess motifs containing mainly alanine and valine with the addition of serine, proline and arginine. However, it should be noted that our cTP data set contained only 16 sequences originating from two species of chlorophytes, so these patterns might not reflect the motifs specific to other Chlorophyta.

Some of the motifs identified in cTPs correspond to sites with experimental evidence of involvement in the import process. For example, LS, L_·_·_S, LSS, L_·_SS, L_·_·_SS, LS_S, L_SS, LSSS, are partial matches to the LLSSS and LKSS motifs reported as critical for protein import to chloroplasts^23^. The abundance and high frequencies of leucine-serine motifs suggest that they may constitute universal motifs for the majority of photosynthetic organisms. Moreover, we found S_S, S_·_SS and SS_·_S present in 71%, 29% and 30% of analysed cTPs, respectively, and they all match the SS_·_SS motif considered to be essential for interaction with Toc159 receptor^22^. The semi-conserved nature of the FGLK motif is the most probable explanation for the absence of this motif in our study; FGLK was proposed to facilitate interaction with Toc34^23,25^.

Mitochondrial transit peptides are the most diverse group of presequences. They do not possess universally conserved motifs, as they have the lowest number of common motifs compared to other presequences. Their motifs are characterized by the presence of leucine, alanine and arginine. Moreover, mTPs display taxon-specific features, especially distinct in the case of plants, which contain many motifs enriched in serine similarly to cTPs. Our results are consistent with previous studies reporting the overrepresentation of arginine as the main difference between mTPs and cTPs^12^, along with the presence of multiple arginine residues and moderately hydrophobic sequence motifs as general determinants of mTPs^26^. Experimental studies suggested φ_·_φφ as a consensus of Tom20-binding motif, where φ corresponds to a hydrophobic or aromatic amino acid^34^. Accordingly, we identified this motif consisting of hydrophobic residues: alanine, valine, isoleucine and leucine in 68% of mTPs. Another work reported VVRNR as one of the essential motifs^22^. Based on the frequent occurrence of motifs such as LR, L_R, L_·_R, LLR and ALR, we suggest that the key feature of this motif is hydrophobicity and the positive charge of arginine, thus valines could probably be replaced by other hydrophobic residues, e.g. leucine or alanine.

SPs represent the most universally spread and well-studied presequences, with the first bioinformatics analyses revealing their three-domain structure performed in the ‘80s^28,35^. Our results show a distinct accumulation of leucine motifs in the central part of SPs, which corresponds well to the hydrophobic h-domain. Although motifs of the h-regions are species-specific, our results show a strong preference for leucine among the majority of SPs^30^. Moreover, we found that plants possess phenylalanine-containing motifs, i.e. FLL, FL_L, L_LFL, whereas fungi are characterized by motifs rich in alanine and sometimes containing serine or valine. Generally, animal SPs have the highest frequency of leucine motifs. However, when analysing animal SPs at a lower taxonomic level, it becomes evident that leucine-rich motifs are more specific to chordates than arthropods. If we go even further, we notice distinct class-specific characteristics. In accordance with previous studies, mammals show the highest number of leucine-rich motifs^30^, but our results provide novel insights about other classes. Thus, arachnids are distinguished by motifs combining leucine and isoleucine, while motifs of insects tend to be composed of leucine, valine and alanine and occur in lower frequencies suggesting higher diversity of their SPs. An interesting trend is observed for SPs of reptiles, which are characterized by many frequently occurring motifs, even of five consecutive amino acids. SPs of snake Kunitz/BPTI inhibitors were shown to be highly conserved and indeed, these sequences comprise a large part of our reptilian data set of SPs^36^.

Even though they are not presequences, antimicrobial peptides display similar physicochemical properties and therefore were hypothesized to give origin to transit peptides. They are characterized by hydrophobicity and positive charge and are enriched mainly in lysine, cysteine and glycine^37^. Considering the motif composition of AMPs and presequences, it does seem more likely that similarities between AMPs and TPs result from convergent evolution rather than common origin. All studied peptides have a high prevalence of hydrophobic amino acids in frequent motifs, especially AMPs and SPs with their lysine and leucine-containing motifs. AMPs and mTPs also share a tendency towards charged residues; however, AMPs show a clear preference for lysine, whereas mTPs for arginine. Taken together, our results do not provide evidence for the support of the antimicrobial origin of TPs.

Our comprehensive study of motifs in presequences and AMPs constitutes a detailed overview of each sequence group, highlighting the similarities and differences between them. Some of the motifs we identified correspond to already known features that were studied experimentally, while others may indicate potentially relevant sites for future experimental research. Considering the functional importance and many practical applications of presequences and AMPs, we believe that our analyses will prove useful for the design, and better understanding of protein import and evolution of targeting peptides.

## Methods

### Data sets

Sequences of proteins containing annotated presequences were downloaded from UniProt 2021_04^38^ using queries provided in Table S1. We chose only those with experimental evidence for presequence annotation. We removed proteins with truncated presequences or unknown cleavage sites. Next, we extracted presequences from each data set by cutting off the N-terminal part according to the annotations. To create final data sets, we discarded presequences containing nonstandard or ambiguous amino acids and shorter than 10. We also removed duplicated sequences. The numbers of sequences at each step of filtering are provided in Table S2. Due to the limited number of available presequences responsible for dual targeting to both chloroplasts and mitochondria, they are not discussed in the article. The numbers of organisms and sequences in each taxonomic group are provided in Table S3.

AMP sequences were downloaded from DBAASP v3.0 database^37^. It contained 15,564 sequences of monomeric AMPs at the time of download. We filtered out sequences containing nonstandard or ambiguous amino acids (3447 sequences), shorter than 10 (1470 sequences) and longer than 100 (17 sequences). Removal of duplicated sequences yielded 9944 peptides suitable for our analyses.

### Motif analysis

All motifs occurring in presequences were extracted using the count_multimers function in seqR R package, supplying the vectors of motif lengths and gap lengths (the number of non-specified residues between specified ones), without counting motif occurrences^39^. We considered motifs ranging from two specified amino acids to five. In order to account for variability and some structural information, we also considered discontinuous motifs with non-specified residues (gaps) between specified amino acids. We selected numbers of known amino acids and gap sizes so that the final length of the considered motif would not exceed 10. For each type of motif, considering its length and distribution of unspecified amino acids, frequencies were calculated as a fraction of presequences that contain a given motif in a given type of sequence and optionally a given taxonomic group. Then, the obtained frequencies of sequences with the given motif were sorted and those representing the most abundant motifs were visualized using ggplot2 R package and saved in HTML reports generated with Rmarkdown; the reports are available in https://doi.org/10.5281/zenodo.8281293 for all types of analyses described below. The density of motif start positions was plotted along the presequences to determine the positional specificity. To simplify the visualizations and interpretation of results, the length of presequences was scaled to 100.

In order to provide evolutionary context of our study, all analyses of presequences were additionally performed for specific taxonomic groups. Differences between motif frequencies among different taxonomic groups were compared using Mann–Whitney test with Benjamini–Hochberg correction for p-value. For insight into more general motifs, all analyses were also carried out using reduced alphabets. To obtain reduced alphabets, amino acids were grouped according to their physicochemical properties into 7 (enc7), 8 (enc8) and 10 (enc10) groups (Table S4).

All individual files corresponding to each type of analysis were investigated manually to extract the most important information. Due to the high computational cost of motif analyses, they could not have been performed automatically for all types at once. The most important information extracted is provided as Supplementary Data. The most frequent motifs available in this file and used for generation of figures were selected using manually selected frequency thresholds, adjusted separately for each type and size of motifs. Additionally, to account for the fact that some motifs may occur multiple times within the same sequence, we performed analyses of multiple occurrences. Here, to reduce the computational cost, only motifs found at least twice in one sequence were considered. This approach allowed analysis of types of motifs collectively according to their length and presence or absence of gaps.

### Supplementary Information

Supplementary Information 1.

Supplementary Information 2.

## Data Availability

All code necessary to reproduce the study is available at https://github.com/ksidorczuk/Presequences-analysis. All protein sequences with accession numbers necessary to reproduce the study and HTML reports generated for individual motifs are available at https://doi.org/10.5281/zenodo.8281293.
