# Supplementary material for: Characterization of signal and transit peptides based on motif composition and taxon-specific patterns
Source: Sci Rep. 2023 Sep 21;13:15751. doi: 10.1038/s41598-023-42987-1 (PMC10514287; doi:10.1038/s41598-023-42987-1)
Supplement: Supplementary file 2 — Supplementary Information 2. [file 41598_2023_42987_MOESM2_ESM.docx]

# Supplementary Information

# Characterization of signal and transit peptides based on motif composition and taxon-specific patterns

Katarzyna Sidorczuk^1^, Paweł Mackiewicz^1^, Filip Pietluch^1^, Przemysław Gagat^1^*

^1^ Department of Bioinformatics and Genomics, Faculty of Biotechnology, University of Wrocław, Poland


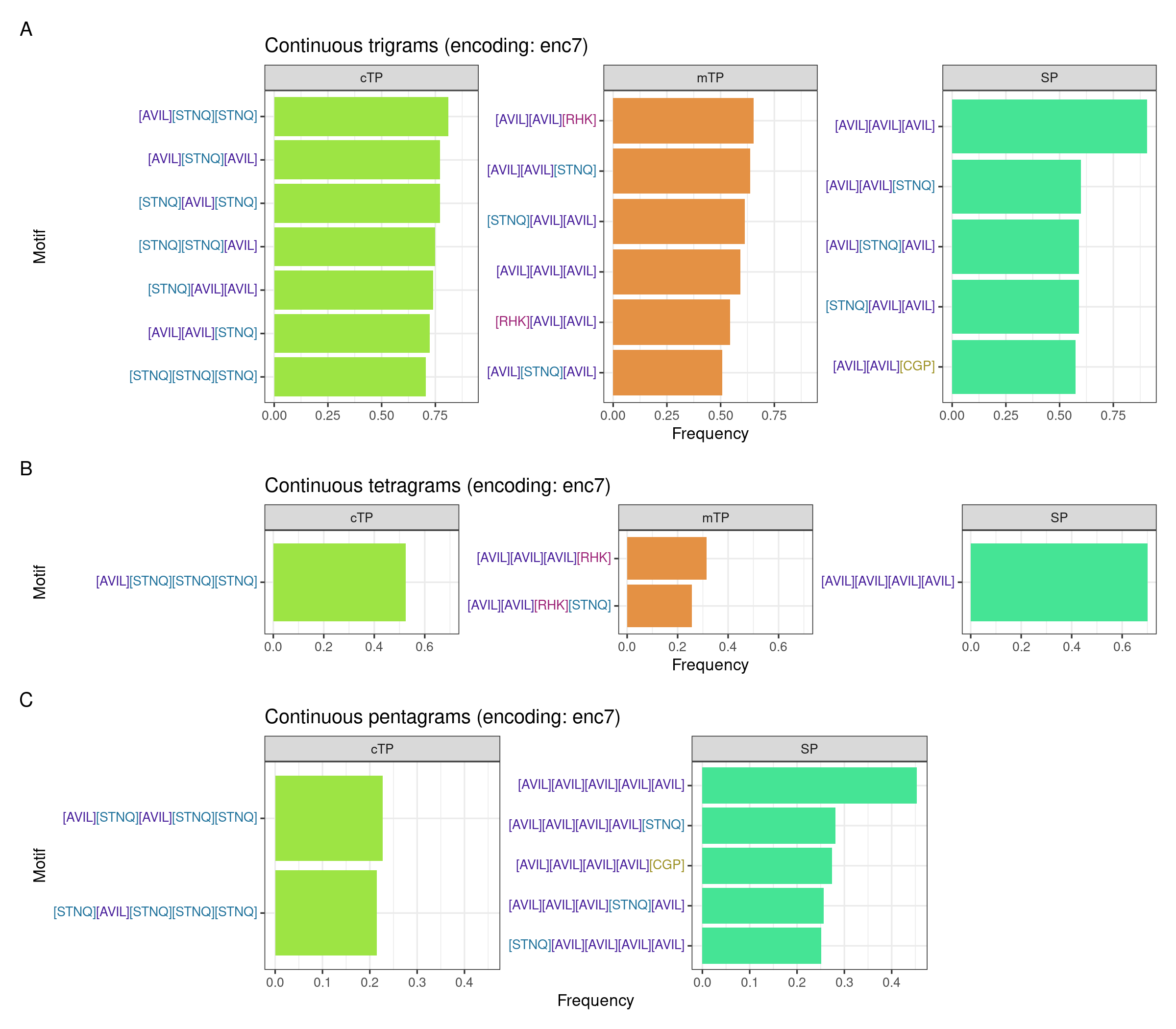


**Figure S1. The most frequent continuous motifs in targeting peptides obtained using enc7 alphabet.** Motifs are presented as regular expressions, where each group of amino acids is shown inside the square bracket and coloured for clarity, indicating that any of these amino acids may be present at this site. Frequency cutoffs have been chosen for each sequence type separately due to the large differences. Data used for plot generation is available in Supplementary Data.


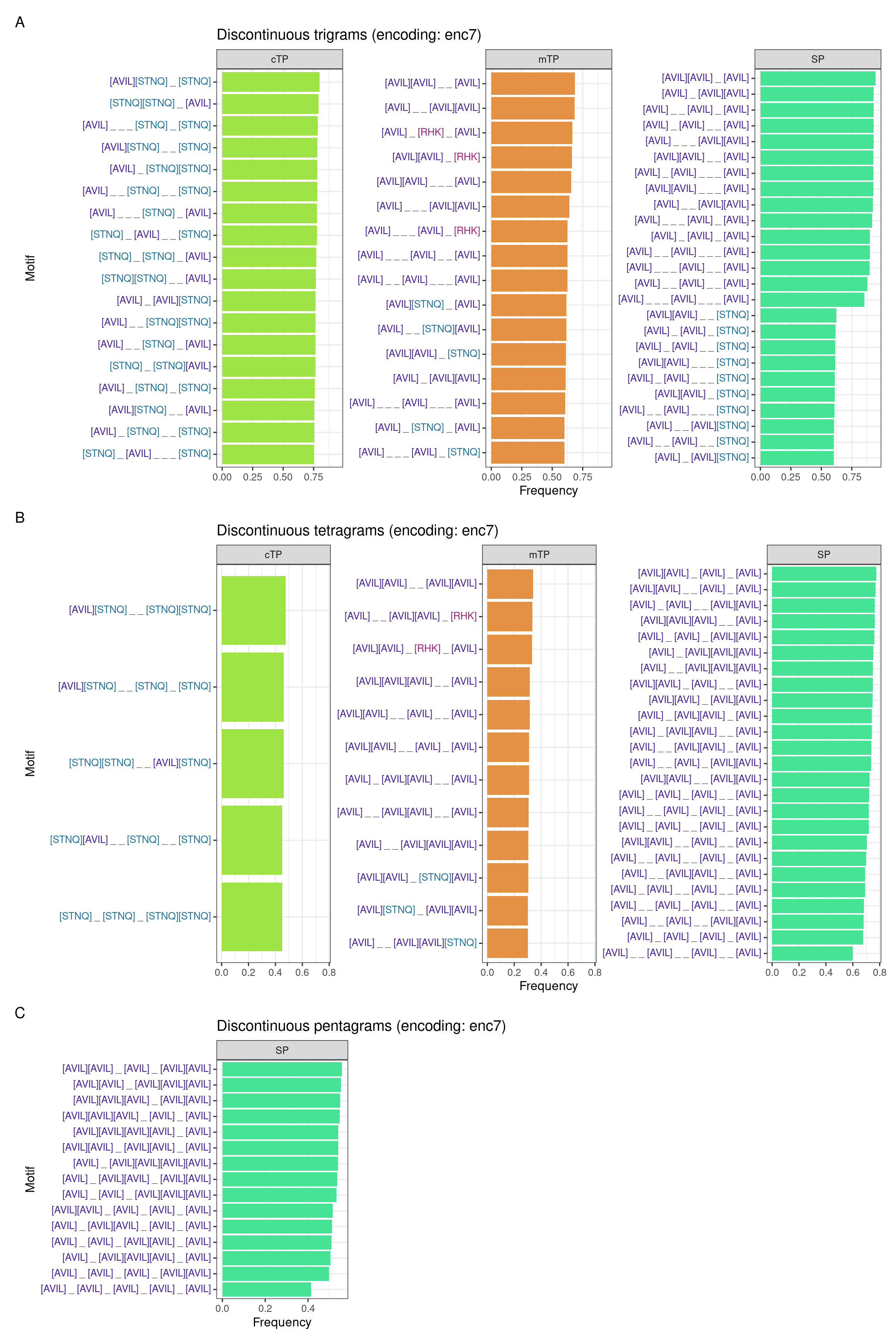


**Figure S2. The most frequent discontinuous motifs in targeting peptides obtained using enc7 alphabet.** Motifs are presented as regular expressions, where each group of amino acids is shown inside the square bracket and coloured for clarity, indicating that any of these amino acids may be present at this location. Frequency cutoffs have been chosen for each sequence type separately due to the large differences. The lower dash symbol ‘_’ indicates an unspecified amino acid in the motif. Data used for plot generation is available in Supplementary Data.


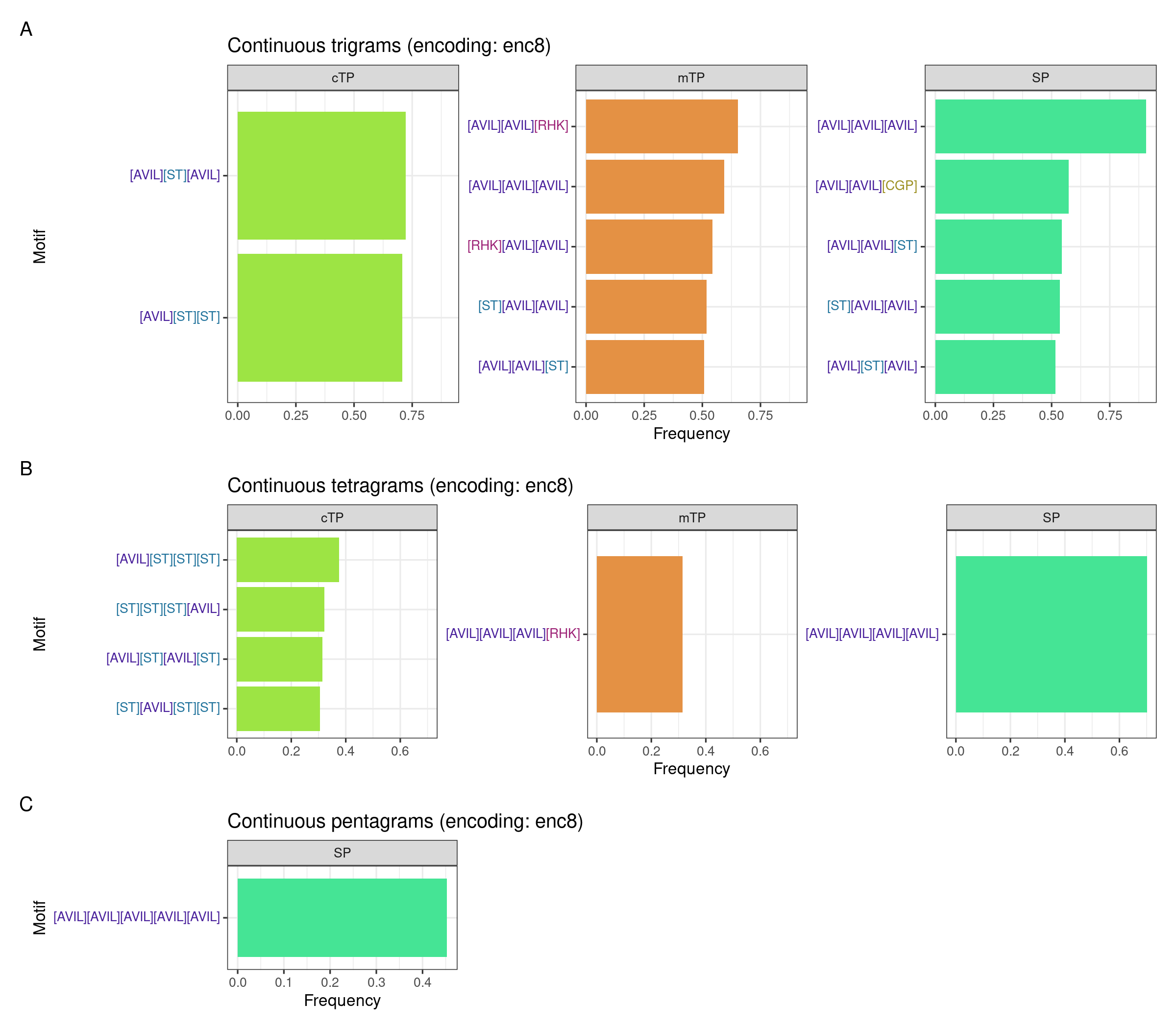


**Figure S3. The most frequent continuous motifs in targeting peptides obtained using enc8 alphabet.** Motifs are presented as regular expressions, where each group of amino acids is shown inside the square bracket and coloured for clarity, indicating that any of these amino acids may be present at this location. Frequency cutoffs have been chosen for each sequence type separately due to the large differences. Data used for plot generation is available in Supplementary Data.

**Figure S4. The most frequent discontinuous motifs in targeting peptides obtained using enc8 alphabet.** Motifs are presented as regular expressions, where each group of amino acids is shown inside the square bracket and coloured for clarity, indicating that any of these amino acids may be present at this location. Frequency cutoffs have been chosen for each sequence type separately due to the large differences. The lower dash symbol ‘_’ indicates an unspecified amino acid in the motif. Data used for plot generation is available in Supplementary Data.


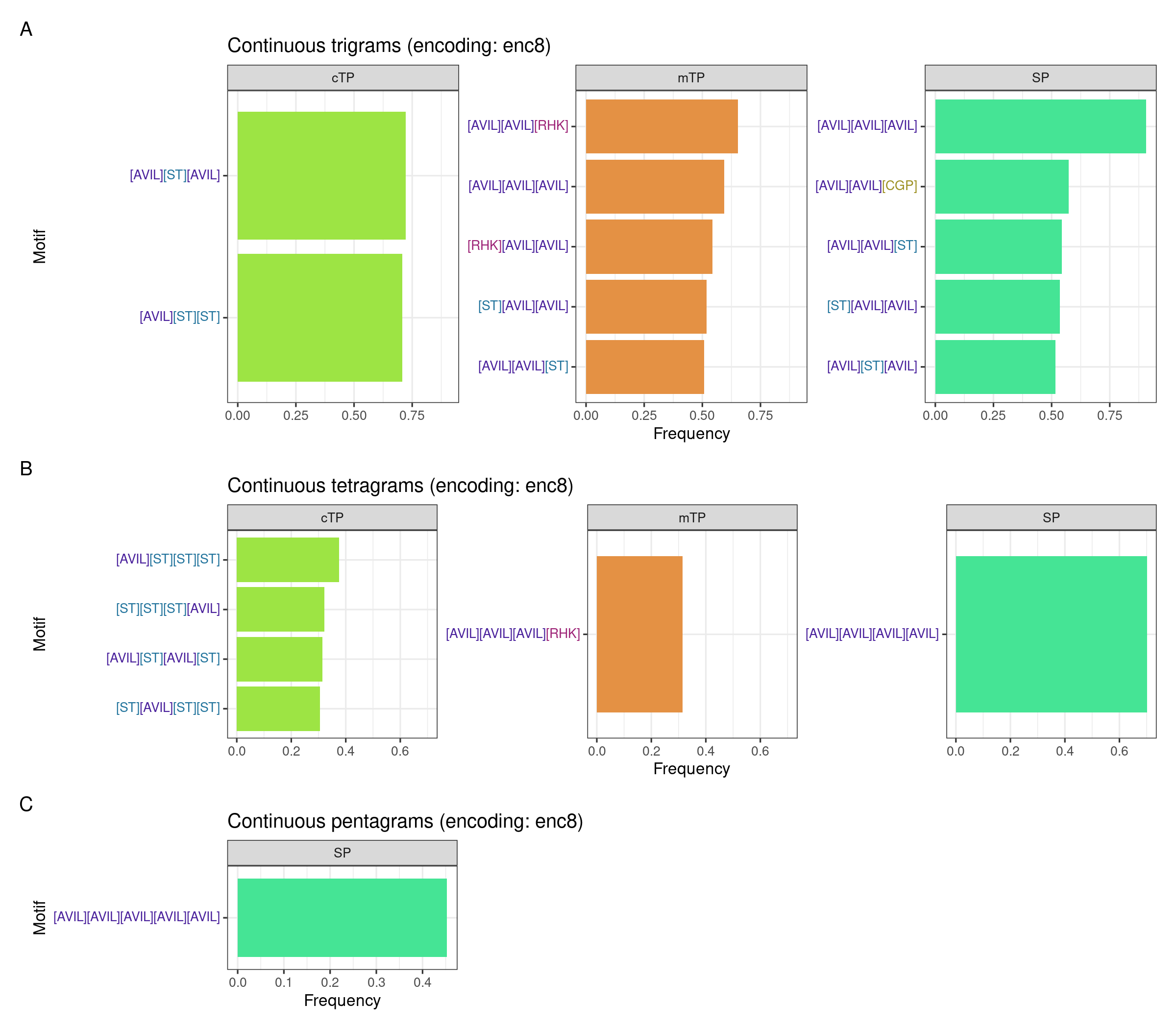

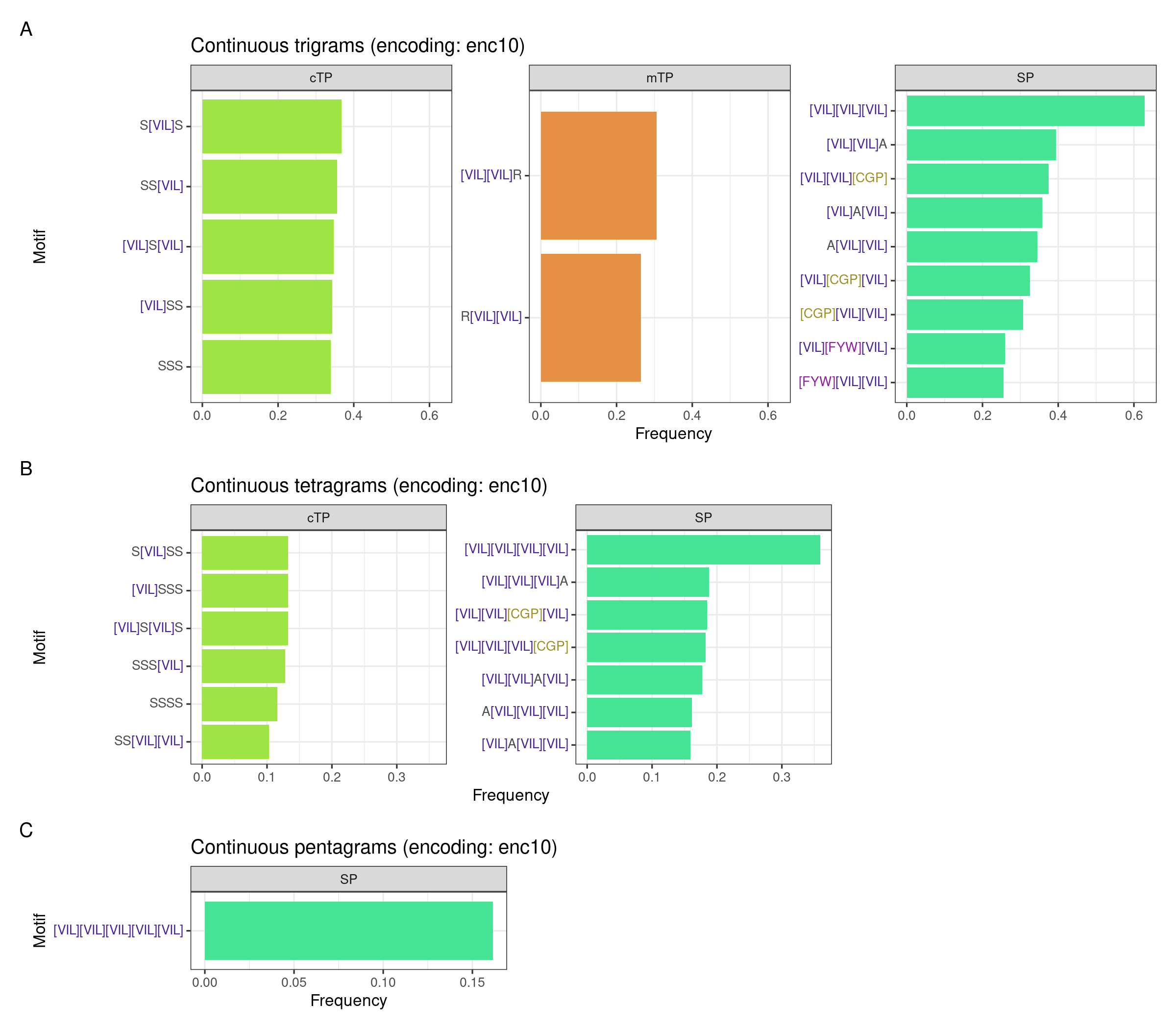


**Figure S5. The most frequent continuous motifs in targeting signals obtained using enc10 alphabet.** Motifs are presented as regular expressions, where each group of amino acids is shown inside the square bracket and coloured for clarity, indicating that any of these amino acids may be present at this location. Frequency cutoffs have been chosen for each sequence type separately due to the large differences. Data used for plot generation is available in Supplementary Data.


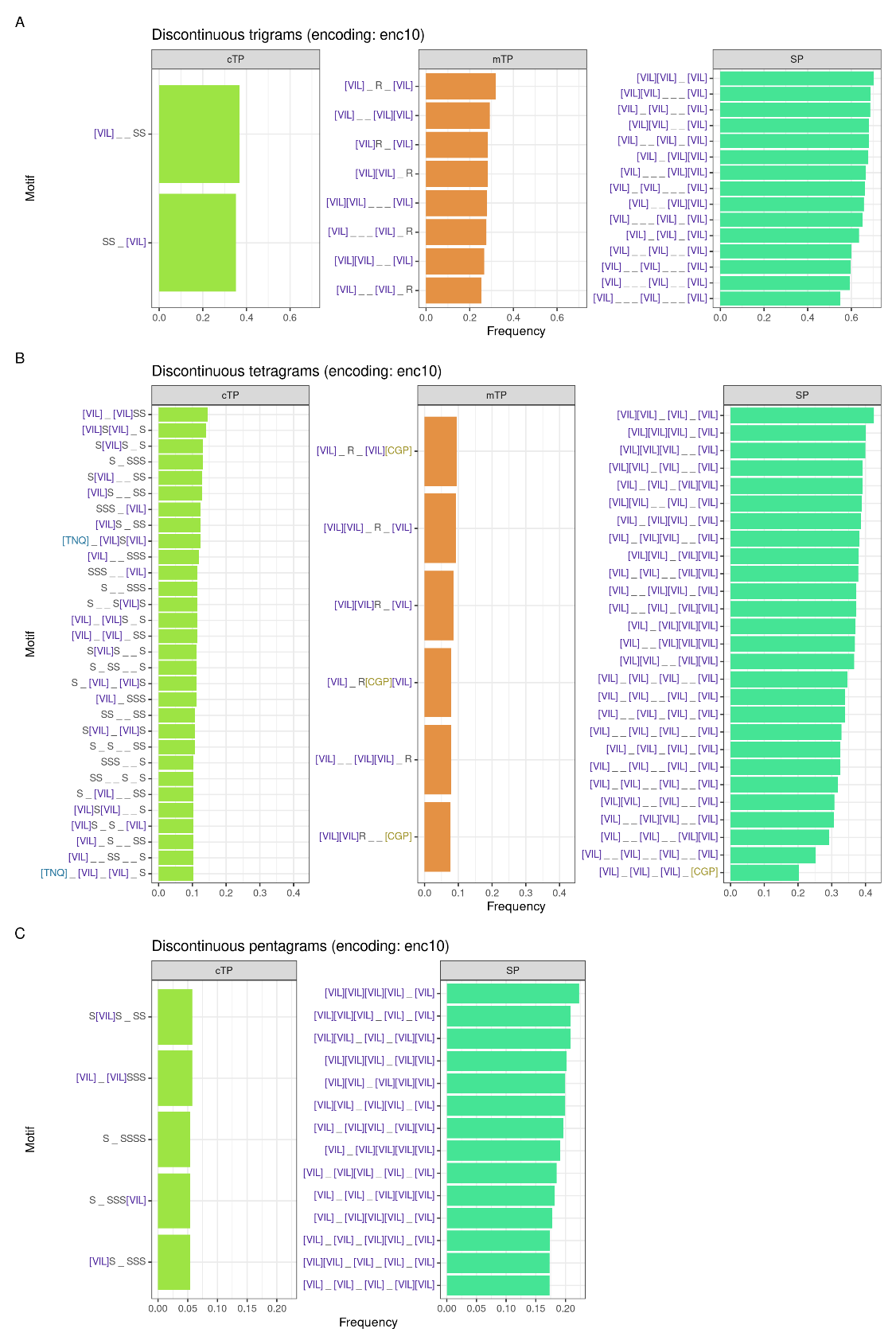


**Figure S6. The most frequent discontinuous motifs in targeting peptides obtained using enc10 alphabet.** Motifs are presented as regular expressions, where each group of amino acids is shown inside the square bracket and coloured for clarity, indicating that any of these amino acids may be present at this location. Frequency cutoffs have been chosen for each sequence type separately due to the large differences. The lower dash symbol ‘_’ indicates an unspecified amino acid in the motif. Data used for plot generation is available in Supplementary Data.


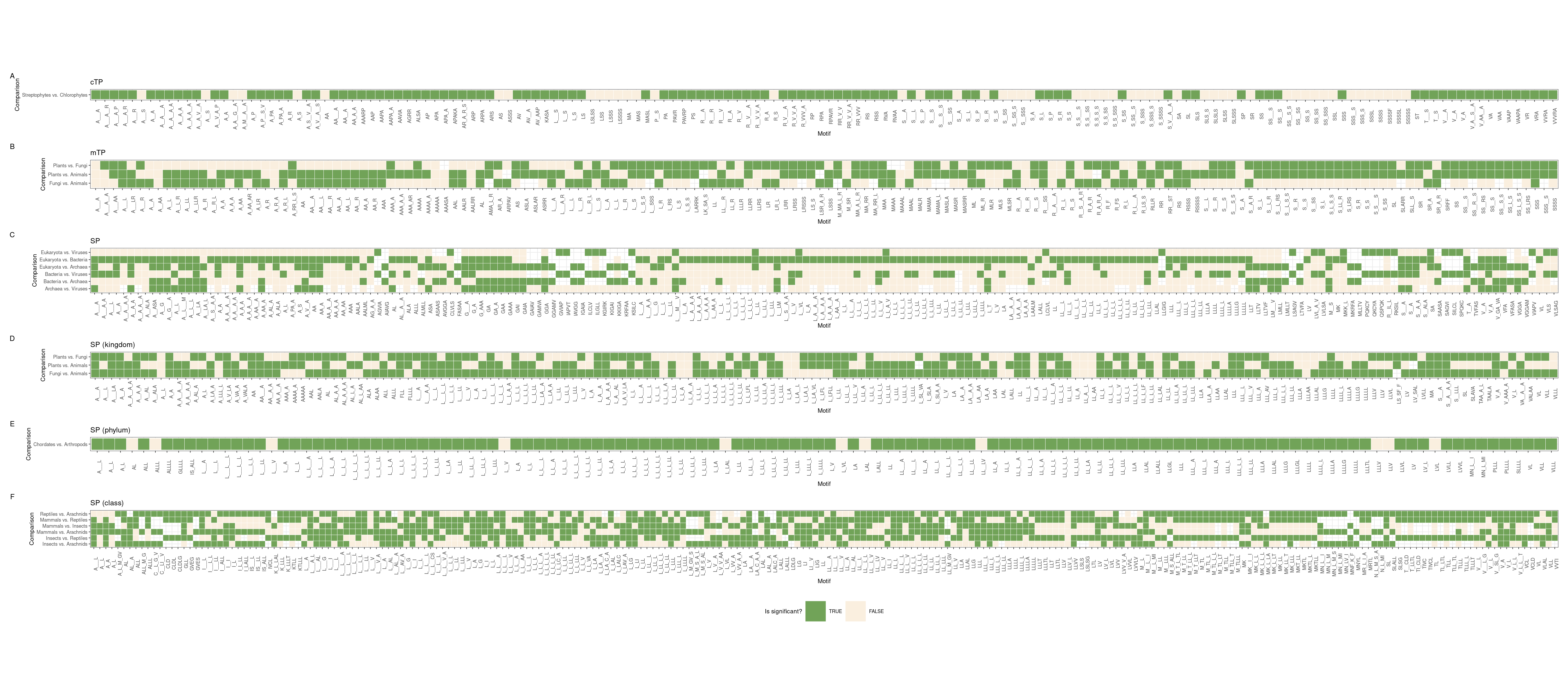


**Figure S7. Statistical analysis of the differences in motif frequency between taxonomic groups.** Motifs are shown on the x-axis and comparisons are indicated on the y-axis. Statistically significant differences with p-value < 0.05 are coloured in green.


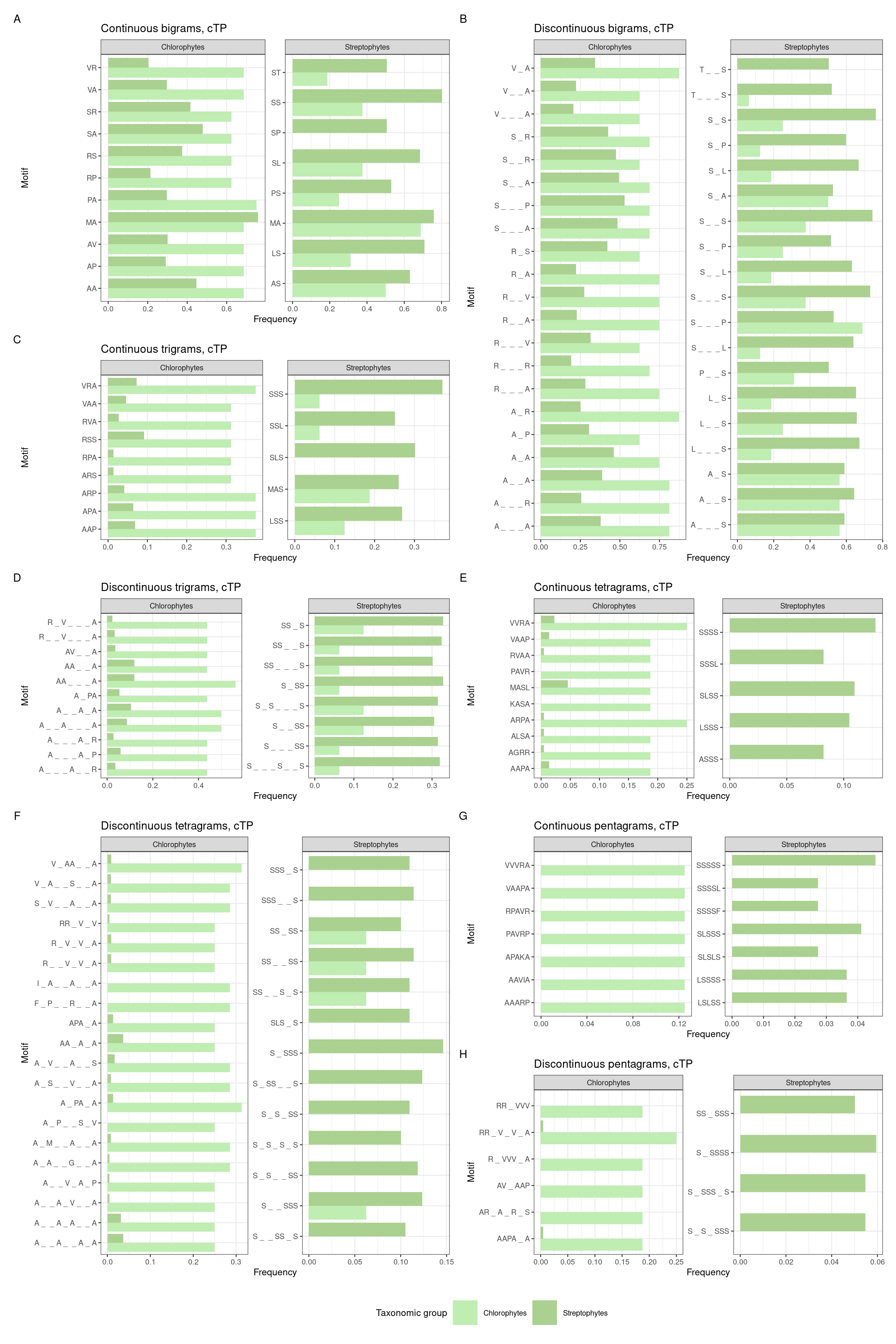


**Figure S8. The most frequent motifs for Streptophyta and Chlorophyta cTPs.** Frequencies are shown for both taxonomic groups for each motif to allow easier comparison. Frequency cutoffs have been chosen for each sequence type separately due to the large differences. The lower dash symbol ‘_’ indicates an unspecified amino acid in the motif. Data used for plot generation is available in Supplementary Data.


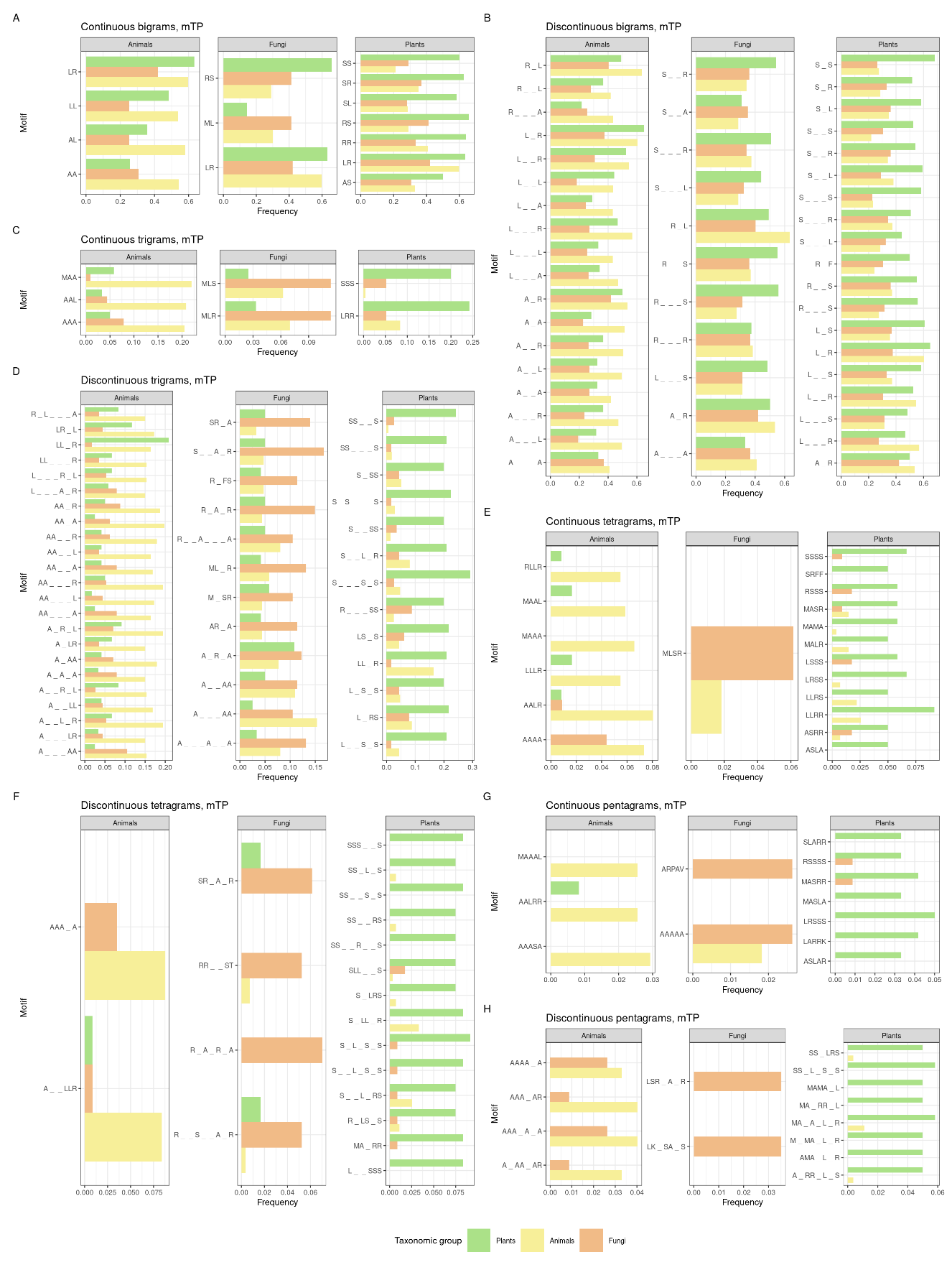


**Figure S9. The most frequent motifs in fungal, animal and plant mTPs.** Frequencies are shown for all taxonomic groups for each motif to allow easier comparison. Frequency cutoffs have been chosen for each sequence type separately due to the large differences. The lower dash symbol ‘_’ indicates an unspecified amino acid in the motif. Data used for plot generation is available in Supplementary Data.


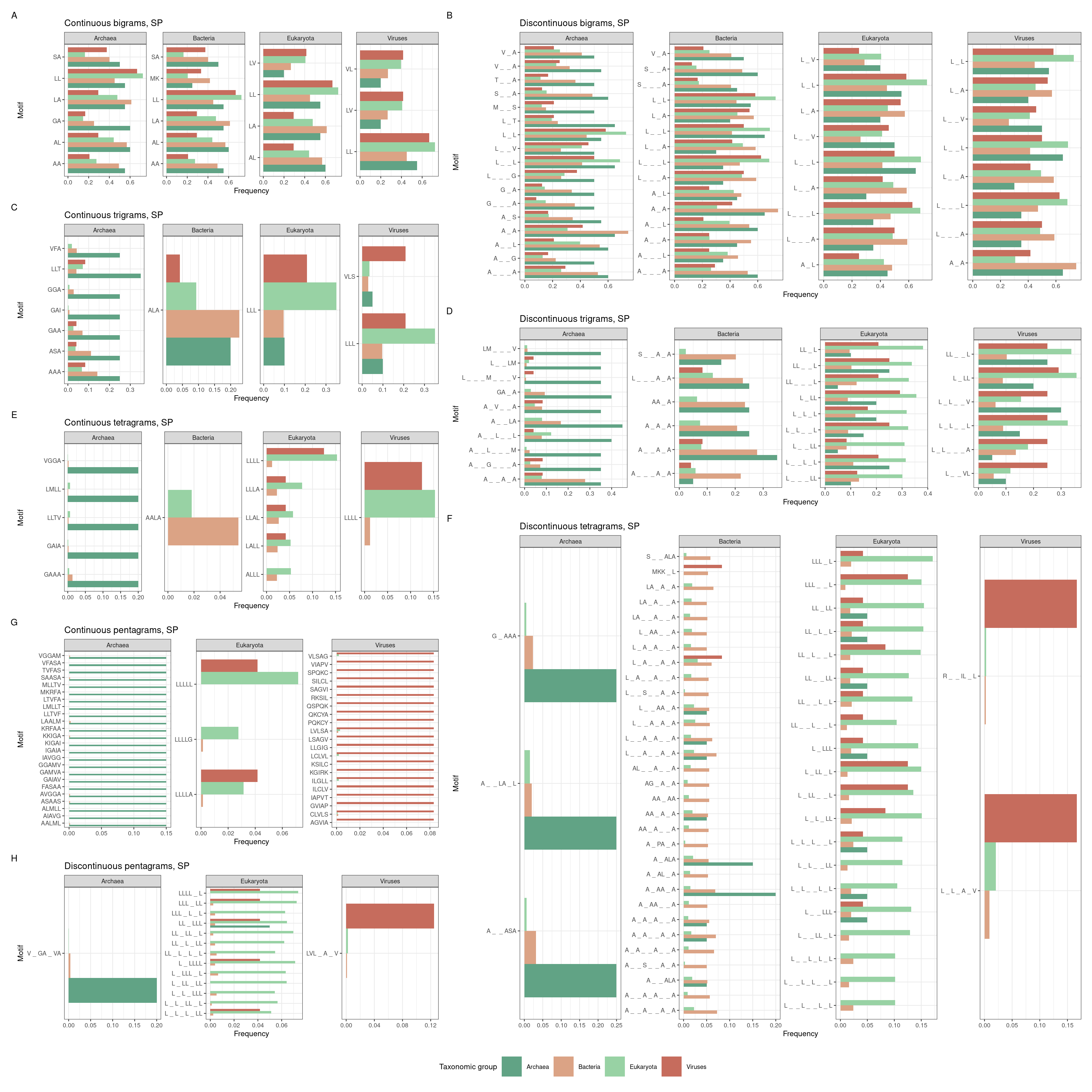


**Figure S10. The most frequent motifs for archaean, bacterial, eukaryotic and viral SPs.** Frequencies are shown for all taxonomic groups for each motif to allow easier comparison. Frequency cutoffs have been chosen for each sequence type separately due to the large differences. The lower dash symbol ‘_’ indicates an unspecified amino acid in the motif. Data used for plot generation is available in Supplementary Data.


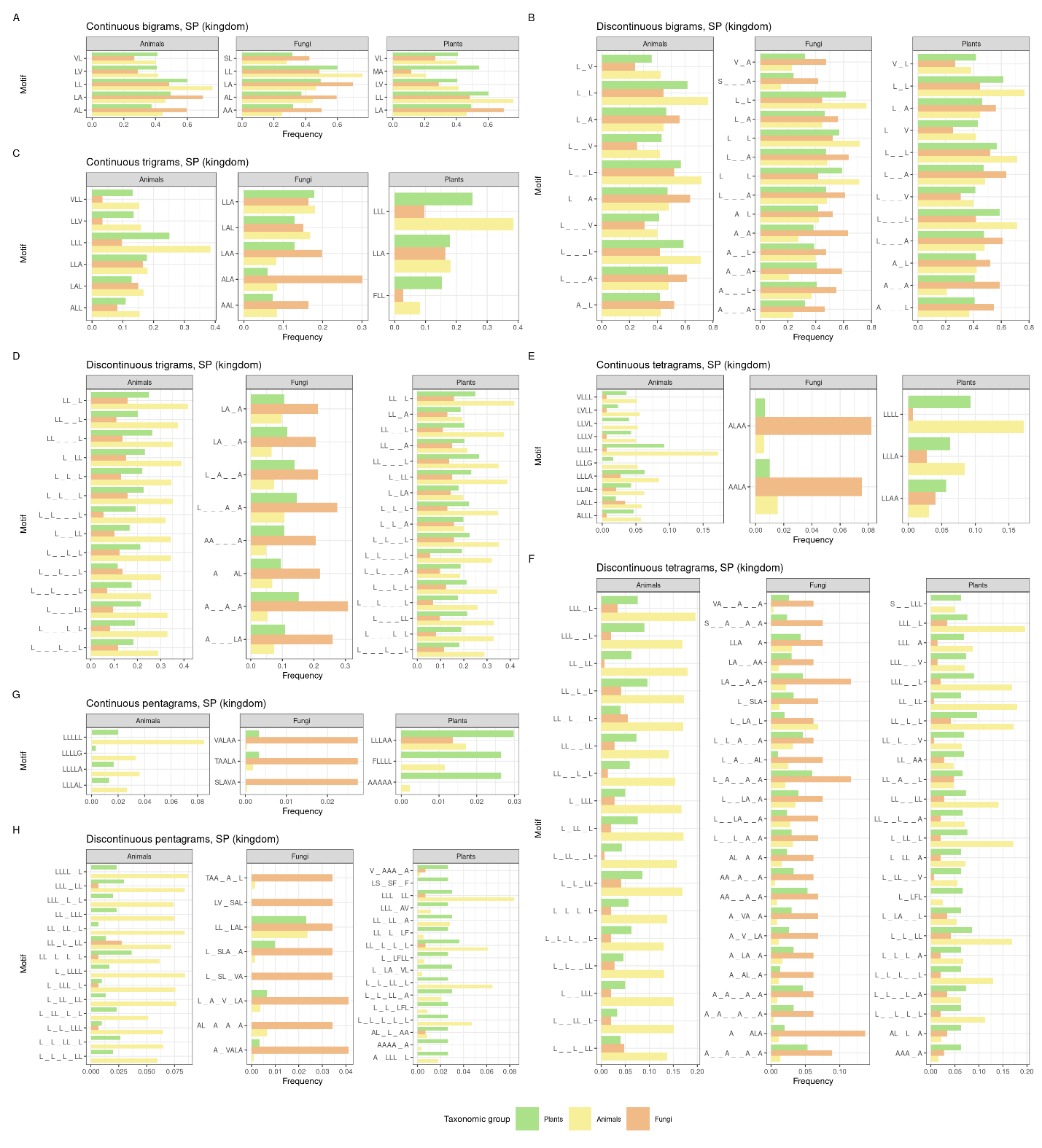


**Figure S11. The most frequent motifs in fungal, animal and plant SPs.** Frequencies are shown for all taxonomic groups for each motif to allow easier comparison. Frequency cutoffs have been chosen for each sequence type separately due to the large differences. The lower dash symbol ‘_’ indicates an unspecified amino acid in the motif. Data used for plot generation is available in Supplementary Data.


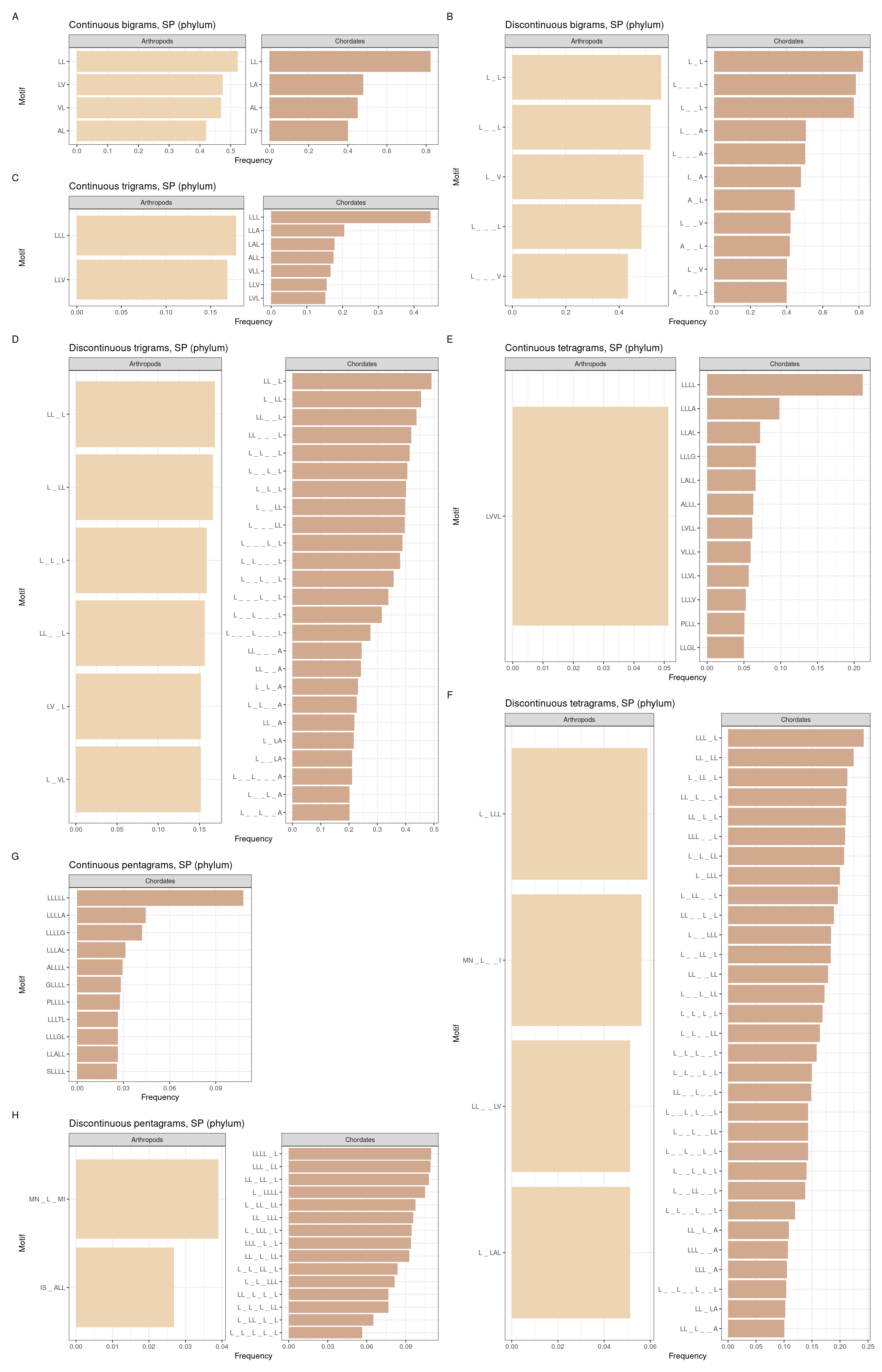


**Figure S12. The most frequent motifs in arthropod and chordate SPs.** Frequencies are shown for all taxonomic groups for each motif to allow easier comparison. Frequency cutoffs have been chosen for each sequence type separately due to the large differences. The lower dash symbol ‘_’ indicates an unspecified amino acid in the motif. Data used for plot generation is available in Supplementary Data.


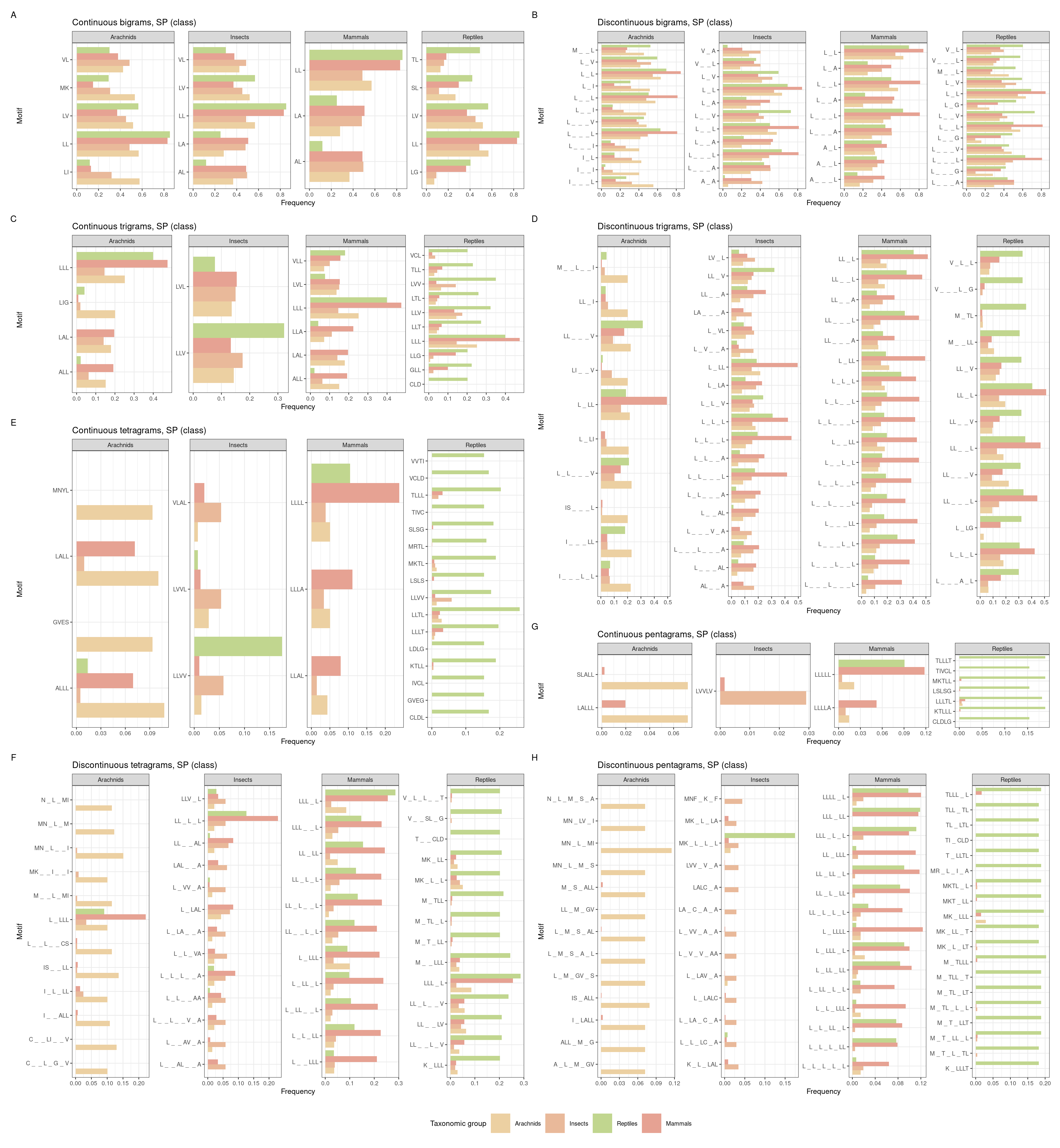


**Figure S13. The most frequent motifs in arachnid, insect, reptile and mammalian SPs.** Frequencies are shown for all taxonomic groups for each motif to allow easier comparison. Frequency cutoffs have been chosen for each sequence type separately due to the large differences. The lower dash symbol ‘_’ indicates an unspecified amino acid in the motif. Data used for plot generation of is available in Supplementary Data.

**Table S1. UniProt queries used to obtain protein sequences with annotated presequences.** cTP – chloroplast transit peptide, mTP – mitochondrial transit peptide, SP – signal peptide, cTP-mTP – transit peptide of proteins dually targeted to plastids and mitochondria.

| Data set | Query |
| --- | --- |
| cTP | locations:(location:"Chloroplast [SL-0049]") annotation:(type:transit evidence:experimental) NOT locations:(location:"Mitochondrion [SL-0173]") |
| mTP | locations:(location:"Mitochondrion [SL-0173]") annotation:(type:transit evidence:experimental) NOT locations:(location:"Chloroplast [SL-0049]") |
| SP | annotation:(type:signal evidence:experimental) |
| cTP-mTP | locations:(location:"Chloroplast [SL-0049]") annotation:(type:transit evidence:experimental) locations:(location:"Mitochondrion [SL-0173]") |

**Table S2. Numbers of sequences in each data set at different steps of processing.** Specifically, numbers of sequences at the time of data downloading (annotated presequence), presequence extraction (annotated presequence location), numbers of presequences with nonstandard or ambiguous amino acids, shorter than 10, identical to others, and the final numbers of presequences used for analyses.

| Data set | With annotated presequence | With annotated presequence location | Containing nonstandard amino acids | Shorter than 10 | Duplicates | To analyse |
| --- | --- | --- | --- | --- | --- | --- |
| cTP | 280 | 246 | 0 | 0 | 4 | 242 |
| mTP | 555 | 549 | 1 | 17 | 10 | 521 |
| SP | 3776 | 3721 | 2 | 1 | 279 | 3439 |
| cTP-mTP | 10 | 9 | 0 | 0 | 0 | 9 |

**Table S3. Numbers of organisms and sequences in each taxonomic group and sequence type**. NA – taxonomic level not available (using the NCBI taxonomy).

| Data  set | Superkingdom | Kingdom | Phylum | Class | Organisms | Sequences |
| --- | --- | --- | --- | --- | --- | --- |
| cTP | Eukaryota | Viridiplantae | Chlorophyta | Chlorophyceae | 2 | 16 |
| cTP | Eukaryota | Viridiplantae | Streptophyta | Magnoliopsida | 27 | 219 |
| cTP | Eukaryota | NA | Euglenozoa | Euglenida | 1 | 3 |
| cTP | Eukaryota | NA | Rhodophyta | Bangiophyceae | 1 | 1 |
| cTP | Eukaryota | NA | Rhodophyta | Florideophyceae | 1 | 1 |
| cTP | Eukaryota | NA | NA | Cryptophyceae | 1 | 1 |
| cTP | Eukaryota | NA | NA | Dinophyceae | 1 | 1 |
| mTP | Eukaryota | Fungi | Ascomycota | Eurotiomycetes | 2 | 3 |
| mTP | Eukaryota | Fungi | Ascomycota | Saccharomycetes | 5 | 93 |
| mTP | Eukaryota | Fungi | Ascomycota | Schizosaccharomycetes | 1 | 2 |
| mTP | Eukaryota | Fungi | Ascomycota | Sordariomycetes | 1 | 15 |
| mTP | Eukaryota | Fungi | Basidiomycota | Tremellomycetes | 1 | 1 |
| mTP | Eukaryota | Metazoa | Arthropoda | Insecta | 2 | 5 |
| mTP | Eukaryota | Metazoa | Chordata | Actinopteri | 2 | 2 |
| mTP | Eukaryota | Metazoa | Chordata | Amphibia | 1 | 2 |
| mTP | Eukaryota | Metazoa | Chordata | Aves | 2 | 7 |
| mTP | Eukaryota | Metazoa | Chordata | Mammalia | 9 | 253 |
| mTP | Eukaryota | Metazoa | Nematoda | Chromadorea | 2 | 4 |
| mTP | Eukaryota | Viridiplantae | Streptophyta | Magnoliopsida | 11 | 120 |
| mTP | Eukaryota | NA | Ciliophora | Oligohymenophorea | 1 | 1 |
| mTP | Eukaryota | NA | Euglenozoa | Euglenida | 1 | 3 |
| mTP | Eukaryota | NA | Euglenozoa | Kinetoplastea | 2 | 7 |
| mTP | Eukaryota | NA | Evosea | Eumycetozoa | 1 | 3 |
| SP | Archaea | NA | Crenarchaeota | Thermoprotei | 3 | 5 |
| SP | Archaea | NA | Euryarchaeota | Archaeoglobi | 1 | 1 |
| SP | Archaea | NA | Euryarchaeota | Halobacteria | 4 | 5 |
| SP | Archaea | NA | Euryarchaeota | Methanobacteria | 1 | 1 |
| SP | Archaea | NA | Euryarchaeota | Methanococci | 3 | 3 |
| SP | Archaea | NA | Euryarchaeota | Methanomicrobia | 4 | 4 |
| SP | Archaea | NA | Euryarchaeota | Thermococci | 1 | 1 |
| SP | Bacteria | NA | Actinobacteria | Actinomycetia | 41 | 68 |
| SP | Bacteria | NA | Aquificae | Aquificae | 2 | 2 |
| SP | Bacteria | NA | Bacteroidetes | Bacteroidia | 3 | 5 |
| SP | Bacteria | NA | Bacteroidetes | Flavobacteriia | 4 | 6 |
| SP | Bacteria | NA | Bacteroidetes | Sphingobacteriia | 3 | 4 |
| SP | Bacteria | NA | Chlamydiae | Chlamydiia | 3 | 4 |
| SP | Bacteria | NA | Chlorobi | Chlorobia | 2 | 2 |
| SP | Bacteria | NA | Chloroflexi | Chloroflexia | 1 | 1 |
| SP | Bacteria | NA | Cyanobacteria | NA | 10 | 20 |
| SP | Bacteria | NA | Deinococcus-Thermus | Deinococci | 3 | 5 |
| SP | Bacteria | NA | Fibrobacteres | Fibrobacteria | 1 | 2 |
| SP | Bacteria | NA | Firmicutes | Bacilli | 58 | 130 |
| SP | Bacteria | NA | Firmicutes | Clostridia | 18 | 23 |
| SP | Bacteria | NA | Proteobacteria | Acidithiobacillia | 3 | 4 |
| SP | Bacteria | NA | Proteobacteria | Alphaproteobacteria | 38 | 57 |
| SP | Bacteria | NA | Proteobacteria | Betaproteobacteria | 20 | 36 |
| SP | Bacteria | NA | Proteobacteria | Deltaproteobacteria | 8 | 16 |
| SP | Bacteria | NA | Proteobacteria | Epsilonproteobacteria | 4 | 7 |
| SP | Bacteria | NA | Proteobacteria | Gammaproteobacteria | 94 | 361 |
| SP | Bacteria | NA | Proteobacteria | Hydrogenophilalia | 1 | 1 |
| SP | Bacteria | NA | Spirochaetes | Spirochaetia | 3 | 3 |
| SP | Bacteria | NA | Tenericutes | Mollicutes | 2 | 2 |
| SP | Eukaryota | Fungi | Ascomycota | Dothideomycetes | 2 | 2 |
| SP | Eukaryota | Fungi | Ascomycota | Eurotiomycetes | 22 | 51 |
| SP | Eukaryota | Fungi | Ascomycota | Pezizomycotina | 3 | 3 |
| SP | Eukaryota | Fungi | Ascomycota | Saccharomycetes | 9 | 39 |
| SP | Eukaryota | Fungi | Ascomycota | Schizosaccharomycetes | 1 | 3 |
| SP | Eukaryota | Fungi | Ascomycota | Sordariomycetes | 15 | 19 |
| SP | Eukaryota | Fungi | Basidiomycota | Agaricomycetes | 11 | 14 |
| SP | Eukaryota | Fungi | Basidiomycota | Microbotryomycetes | 1 | 1 |
| SP | Eukaryota | Fungi | Basidiomycota | Tremellomycetes | 2 | 3 |
| SP | Eukaryota | Fungi | Basidiomycota | Ustilaginomycetes | 2 | 2 |
| SP | Eukaryota | Fungi | Chytridiomycota | Neocallimastigomycetes | 1 | 2 |
| SP | Eukaryota | Fungi | Microsporidia | NA | 2 | 3 |
| SP | Eukaryota | Fungi | Mucoromycota | Mucoromycetes | 3 | 4 |
| SP | Eukaryota | Metazoa | Annelida | Clitellata | 6 | 9 |
| SP | Eukaryota | Metazoa | Annelida | Polychaeta | 1 | 4 |
| SP | Eukaryota | Metazoa | Arthropoda | Arachnida | 58 | 139 |
| SP | Eukaryota | Metazoa | Arthropoda | Chilopoda | 4 | 26 |
| SP | Eukaryota | Metazoa | Arthropoda | Collembola | 1 | 1 |
| SP | Eukaryota | Metazoa | Arthropoda | Insecta | 57 | 206 |
| SP | Eukaryota | Metazoa | Arthropoda | Malacostraca | 14 | 21 |
| SP | Eukaryota | Metazoa | Arthropoda | Merostomata | 2 | 15 |
| SP | Eukaryota | Metazoa | Arthropoda | Thecostraca | 1 | 1 |
| SP | Eukaryota | Metazoa | Chordata | Actinopteri | 26 | 52 |
| SP | Eukaryota | Metazoa | Chordata | Amphibia | 9 | 26 |
| SP | Eukaryota | Metazoa | Chordata | Ascidiacea | 1 | 1 |
| SP | Eukaryota | Metazoa | Chordata | Aves | 7 | 51 |
| SP | Eukaryota | Metazoa | Chordata | Chondrichthyes | 2 | 4 |
| SP | Eukaryota | Metazoa | Chordata | Hyperoartia | 1 | 2 |
| SP | Eukaryota | Metazoa | Chordata | Lepidosauria | 66 | 143 |
| SP | Eukaryota | Metazoa | Chordata | Mammalia | 35 | 1378 |
| SP | Eukaryota | Metazoa | Chordata | Myxini | 1 | 1 |
| SP | Eukaryota | Metazoa | Chordata | NA | 1 | 1 |
| SP | Eukaryota | Metazoa | Cnidaria | Anthozoa | 4 | 4 |
| SP | Eukaryota | Metazoa | Cnidaria | Cubozoa | 1 | 2 |
| SP | Eukaryota | Metazoa | Echinodermata | Asteroidea | 2 | 3 |
| SP | Eukaryota | Metazoa | Mollusca | Bivalvia | 8 | 17 |
| SP | Eukaryota | Metazoa | Mollusca | Gastropoda | 14 | 32 |
| SP | Eukaryota | Metazoa | Nematoda | Chromadorea | 5 | 14 |
| SP | Eukaryota | Viridiplantae | Chlorophyta | Chlorophyceae | 1 | 4 |
| SP | Eukaryota | Viridiplantae | Streptophyta | Magnoliopsida | 117 | 291 |
| SP | Eukaryota | Viridiplantae | Streptophyta | Pinopsida | 6 | 7 |
| SP | Eukaryota | NA | Apicomplexa | Aconoidasida | 1 | 1 |
| SP | Eukaryota | NA | Euglenozoa | Kinetoplastea | 2 | 3 |
| SP | Eukaryota | NA | Evosea | Eumycetozoa | 3 | 19 |
| SP | Eukaryota | NA | Evosea | NA | 1 | 6 |
| SP | Eukaryota | NA | Haptophyta | NA | 1 | 1 |
| SP | Eukaryota | NA | Oomycota | NA | 3 | 3 |
| SP | Eukaryota | NA | Rhodophyta | Bangiophyceae | 1 | 1 |
| SP | Eukaryota | NA | Rhodophyta | Florideophyceae | 1 | 1 |
| SP | Viruses | Bamfordvirae | Nucleocytoviricota | Pokkesviricetes | 1 | 1 |
| SP | Viruses | Bamfordvirae | Preplasmiviricota | Tectiliviricetes | 2 | 3 |
| SP | Viruses | Heunggongvirae | Peploviricota | Herviviricetes | 3 | 3 |
| SP | Viruses | Heunggongvirae | Uroviricota | Caudoviricetes | 4 | 4 |
| SP | Viruses | Loebvirae | Hofneiviricota | Faserviricetes | 3 | 4 |
| SP | Viruses | Orthornavirae | Duplornaviricota | Chrymotiviricetes | 1 | 1 |
| SP | Viruses | Orthornavirae | Duplornaviricota | Resentoviricetes | 1 | 1 |
| SP | Viruses | Orthornavirae | Negarnaviricota | Ellioviricetes | 2 | 2 |
| SP | Viruses | Orthornavirae | Negarnaviricota | Monjiviricetes | 1 | 1 |
| SP | Viruses | Orthornavirae | Pisuviricota | Pisoniviricetes | 1 | 1 |
| SP | Viruses | Pararnavirae | Artverviricota | Revtraviricetes | 3 | 3 |

**Table S4. Groups of amino acids corresponding to reduced alphabets used in the study.**

| Alphabet (encoding name) | Amino acid groups |
| --- | --- |
| enc10 | S |
|  | T, N, Q |
|  | A |
|  | V, I, L |
|  | M |
|  | F, Y, W |
|  | C, G, P |
|  | D, E |
|  | R |
|  | H, K |
| enc8 | S, T, N, Q |
|  | A, V, I, L |
|  | M |
|  | F, Y, W |
|  | C, G, P |
|  | D, E |
|  | R, H, K |
| enc7 | S, T |
|  | N, Q |
|  | A, V, I, L |
|  | M |
|  | F, Y, W |
|  | C, G, P |
|  | D, E |
|  | R, H, K |
